# Opioid Use Disorder Curriculum: Medicine Clerkship Standardized Patient Case, Small-Group Activity, and Patient Panel

**DOI:** 10.15766/mep_2374-8265.11248

**Published:** 2022-05-24

**Authors:** Hansel E. Tookes, Jasmine Tomita-Barber, Sabrina Taldone, Morgan Shane, Matthew R. Imm, Henri Ford, Joan St. Onge, David W. Forrest, Tyler S. Bartholomew, David P. Serota

**Affiliations:** 1 Associate Professor of Clinical Medicine, Department of Medicine, University of Miami Leonard M. Miller School of Medicine; 2 First-Year Resident, Department of Medicine, University of Miami Leonard M. Miller School of Medicine; 3 Assistant Professor of Clinical Medicine, Department of Medicine, University of Miami Leonard M. Miller School of Medicine; 4 Dean and Chief Academic Officer, University of Miami Leonard M. Miller School of Medicine; 5 Professor of Clinical Medicine, Department of Medicine, University of Miami Leonard M. Miller School of Medicine; 6 Research Associate Professor, Department of Public Health Sciences, University of Miami Leonard M. Miller School of Medicine; 7 Research Assistant Professor, Department of Public Health Sciences, University of Miami Leonard M. Miller School of Medicine

**Keywords:** Substance Abuse/Addiction, Clinical Skills Assessment/OSCEs, Standardized Patient, Opioids, Addiction, Pain

## Abstract

**Introduction:**

The overdose crisis remains a critical public health problem, creating an urgent need to train physicians in the treatment and management of opioid use disorder (OUD). Our medicine clerkship module aimed to close this gap by training and assessing students’ motivational interviewing skills, harm reduction knowledge, and use of nonstigmatizing language in the treatment of patients with OUD.

**Methods:**

We evaluated the impact of a small-group, case-based activity and patient panel on the clinical documentation skills of students in a medicine clerkship. Clinical documentation was based on an observed structured clinical examination of a standardized patient with OUD and was evaluated using a grading rubric that followed the module learning objectives. Students also submitted reflections on the curriculum.

**Results:**

Qualitative responses (*n* = 40) from students evaluating the small-group activity and patient panel exercise revealed overall student satisfaction with the patient panel and exposure to patients living with OUD. Three themes emerged from student reflections: (1) humanity, (2) different paths to recovery, and (3) using nonstigmatizing language. For the quantitative test, students’ (*n* = 39) mean clinical documentation scores before and after the small-group activity and patient panel increased from 10.1 to 11.3 out of 13.5 possible points. There was a significant difference between mean pretest and posttest scores (*p* < .001).

**Discussion:**

The medicine clerkship provided an acceptable and feasible opportunity for implementing a multifaceted educational experience for students with significant immediate impact on their evaluation of patients with OUD.

## Educational Objectives

By the end of this activity, learners will be able to:

1. Record relevant patient history in a patient with opioid use disorder (OUD).
2. Revise word choice to avoid stigmatizing language in clinical documentation for patients with OUD.
3. Recommend treatment options for OUD.
4. Propose harm reduction strategies in patients with higher-risk behaviors due to OUD.

## Introduction

The overdose crisis remains a critical public health problem, creating an urgent need to train physicians in the treatment and management of opioid use disorder (OUD).^1,2^ According to the 2019 National Survey on Drug Use and Health, 1.6 million people are living with OUD, and of those, only 18% received medications for OUD (MOUD) in the past year.^2^ Inability to obtain MOUD can be due to a number of complex obstacles, including financial barriers, stigma, arduous treatment regulations, and lack of access to physicians with a Drug Enforcement Administration (DEA) waiver.^2–4^ While increasing availability of MOUD is critical to addressing the overdose crisis, many physicians underutilize these lifesaving medications. Among the barriers to prescribers are a lack of sufficient training, education, and experience in prescribing buprenorphine, and provider stigma.^5–9^

With the passage of the Substance Use-Disorder Prevention That Promotes Opioid Recovery and Treatment (SUPPORT) for Patients and Communities Act into law, physicians are eligible for a DEA waiver if they graduate in good standing from an allopathic or osteopathic medical school that provides a comprehensive education on management of OUD.^10^ This legislation offers an opportunity to train new generations of physicians on how to care for patients with OUD with competency and compassion.^11^ Brown University's Warren Alpert Medical School was the first to develop a comprehensive curriculum on OUD designed for medical students. With the passing of the SUPPORT for Patient and Communities Act and the approval of the curriculum by the Substance Abuse and Mental Health Services Administration (SAMHSA), Brown's medical students will be automatically eligible to apply for a waiver to prescribe MOUD upon graduation.^11,12^

Educators at the University of Miami Leonard M. Miller School of Medicine, funded by the Florida SAMHSA recipient, the Department of Children and Families, have sought to create a curriculum that will provide medical students with the skills necessary to assess, diagnose, and treat substance use disorder (SUD) in general, and OUD specifically, prior to graduation. Herein, we describe an objective structured clinical examination (OSCE) designed for third-year medical students during their medicine clerkship. An OSCE was the exercise of choice as it is considered a gold standard for assessing clinical competency among students and represents the third (i.e., shows how) level of Miller's pyramid of assessment.^13,14^ The purpose of the OSCE was to have students practice motivational interviewing with a standardized patient (SP) living with OUD. By engaging in this activity, students could practice motivational interviewing and counseling while being cognizant of using person-first language and avoiding stigmatizing labels. The OSCE was designed as a low-stakes, formative exercise. Students also participated in a small-group, case-based discussion followed immediately by a patient panel with people living with OUD. This panel was intended to be a humanizing experience, allowing students to reflect on their perceptions and ideas surrounding this marginalized population.

To our knowledge, this is the only exercise of its kind specifically designed for medical students in a medicine clerkship. In an interprofessional workshop at the Warren Alpert Medical School, students participated in an SP (simulation) case: Students were asked to obtain a history; perform a brief, focused physical examination; and demonstrate the appropriate use of the SBIRT (screening, brief intervention, referral for treatment) approach. Faculty observers then provided verbal feedback to students. However, the authors of the report describing that workshop did not include a direct analysis of the SP intervention.^15^ Other published OSCEs are either intended for residents or focused on identification of OUD and communication skills during a difficult patient encounter.^16,17^ In contrast, our exercise emphasizes motivational interviewing, harm reduction, HIV/hepatitis C virus (HCV) prevention, and use of nonstigmatizing language.

## Methods

In 2019, faculty at the University of Miami Leonard M. Miller School of Medicine received a state opioid response grant funded by the Florida Department of Children and Families to implement an OUD curriculum in undergraduate medical education. The goal was to include elements of OUD, SUD, and harm reduction education in existing aspects of the curriculum. We sought to increase students’ knowledge and skills regarding these traditionally overlooked topics by exposing students to them multiple times over their education. Using the grant, we developed educational tools, including a case-based discussion on opioid pharmacology for the basic science curriculum^18^; a case of medication treatment of OUD and harm reduction for the neuroscience unit; and the OSCE, case-based discussion, and patient panel for the medicine clerkship described in this report.

### OSCE

Prior to the exercise, we recommended that students read a review article and listen to a podcast.^19,20^ Students completed a case-based learning activity on SUD during their preclinical studies, but no didactic or other preparation was provided directly before the OSCE. There was no formal assessment of whether students had completed the prework. Students were provided with instructions for the OSCE (Appendix A). The OSCE was designed for the knowledge and skill level of third-year medical students in the medicine clerkship at the University of Miami Leonard M. Miller School of Medicine. The goal of the OSCE was to have students apply motivational interviewing skills to a patient with OUD and to deliver counseling on OUD and HIV/HCV screening and prevention. The OSCE was designed to promote the Association of American Medical Colleges’ core entrustable professional activities (EPAs) of gathering a history, prioritizing a differential diagnosis, and documenting a clinical encounter (EPAs 1, 2, and 5, respectively).^21^ The OSCE case concerned a young patient presenting to clinic and requesting help to stop using opioids. Logistically, each session consisted of 20 minutes for the in-person SP encounter, 5 minutes for feedback from the SP, and 20 minutes to document the encounter in the format of a history and physical. Students were also expected to demonstrate empathy and use appropriate nonstigmatizing language when communicating with the patient and documenting the encounter. Students were asked to submit clinical documentation of the OSCE before completing the case-based discussion and patient panel sessions. Following those sessions, students were given their original documentation and had the opportunity to make edits based on the case and patient panel experiences.

All activities took place in the Gordon Center for Simulation and Innovation in Medical Education during a full day of didactics in the medicine clerkship. The primary faculty developers of the OSCE were authors Hansel E. Tookes, Sabrina Taldone, and David P. Serota—the co-course directors of the integrated OUD curriculum—in iterative consultation with peers who worked at the IDEA Syringe Services Program (SSP), our local harm reduction program. SPs recruited included young to middle-age adults. SPs were not involved with the design of the OSCE. SPs were trained by Gordon Center staff in a session during which the case was reviewed and all SP questions were answered. The script can be found in Appendix B. At the end of each student's OSCE, SPs provided verbal formative feedback on student performance was based on an SP checklist guideline/feedback script (Appendix C).^22^ SPs were instructed to give yes/no responses based on the evaluation questions in Appendix C and told not to give partial credit. Six SPs saw six to seven students each during the morning exercise. The SPs debriefed in a “de-role” guided meditation during which they shed their role as the OSCE patient with OUD and discussed their positive and negative experiences from the session.

### Small-Group, Case-Based Activity and Patient Panel

After the OSCE, students participated in a 1-hour, small-group, case-based exercise (Appendix D, the student version of the case, and Appendix E, the facilitator version of the case) centering on a patient with OUD. To ensure that the case was culturally appropriate, our team consulted with people with lived experience from our university's SSP throughout the development of the case. Important themes from the small-group activity were buttressed by the patient panel. This small-group activity, delivered via videoconference, was written with a harm reduction focus and facilitated by one instructor who shifted between breakout rooms during the videoconference and convened the entire group to discuss responses. The goal of the small-group discussion was to facilitate analytical student thinking about OUD, harm reduction, and how stigma impacts the medical care of people with OUD, especially in the hospital. The case served to set up the patient panel. Objectives covered in the small-group case included diagnosis and treatment of opioid overdose, urine drug screen interpretation, treating pain in patients hospitalized with OUD, decision-making in managing injection drug use–associated infections, and harm reduction (Appendices D and E).

The activity was followed by a 1-hour patient panel with two to three patients with OUD who were recruited from the local SSP. These panels were moderated by a physician with expertise in treating patients with OUD. The patients were asked a standardized set of questions about their recovery experience:

• What does recovery mean to you?
• What medications for OUD—if any—are you taking and why?
• How has stigma against people who use drugs impacted your experience with health care?

The patients were all in different stages of recovery, and after they had illustrated their recovery journey, the students were provided the opportunity to ask them questions. Finally, the students were asked to revise their OSCE documentation with the aim of removing stigmatizing language and using person-first language. A session flow diagram is included in the Figure.

**Figure.**
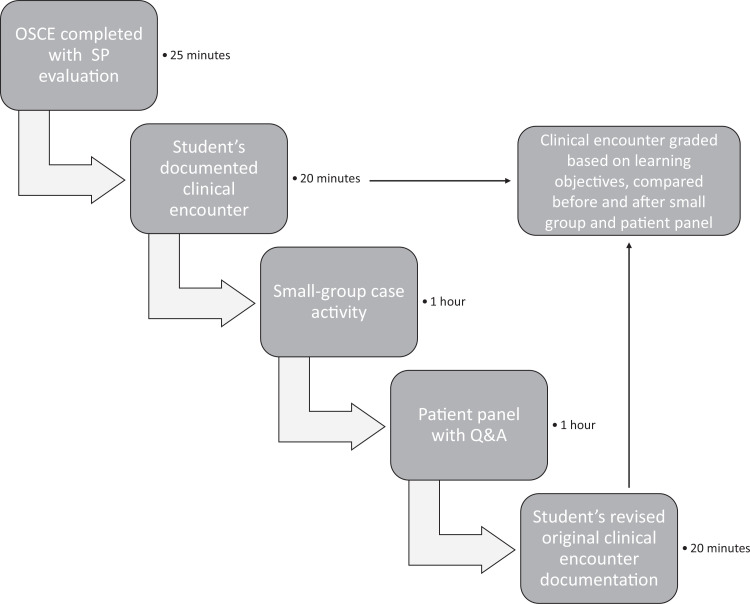
Opioid use disorder and harm reduction activity flow diagram. This medicine clerkship flow diagram shows the various steps involved in the curriculum and the time needed for each component. Abbreviations: OSCE, objective structured clinical examination; SP, standardized patient.

### Evaluation

We utilized a mixed-methods evaluation to examine the impact of the small-group, case-based activity and patient panel. At the time of the study, the medicine clerkship ran for 8 weeks and typically included about 40 students in each cohort. First, we pilot-tested our small-group activity/patient panel component prior to our pre/post clinical documentation evaluation. The first cohort of students was asked to “briefly describe how [their] opinions about people with OUD changed or did not change as a result of the patient panel discussion.” Responses were analyzed using a general inductive approach.^23^ Two authors (Hansel E. Tookes and Tyler S. Bartholomew) read all responses and condensed the transcript data into summary statements. Emerging themes from the interviews were presented to the entire team and used to evaluate student acceptance of and satisfaction with the small-group activity and patient panel during the pilot-testing phase. Although the case-based, small-group activity was not specifically evaluated, it took place immediately before the patient panel and served as an introduction to its themes.

Next, with a second cohort of medicine clerkship students, two masked members of the research team independently reviewed and graded the student clinical documentation before and after the small group/patient panel using a standardized rubric (Appendix F). The grading rubric included assessment of Educational Objectives 1, 2, and 3. Educational Objective 4 was not directly measured. The difference between the mean scores before and after the small group/patient panel was examined using a paired samples *t* test. To avoid the problem of multiple comparisons in statistical testing, we compared only total scores before and after revision, not differences on individual items. Our scoring rubric weighted items. The most highly weighted items focused on completion of all parts of the note and avoidance of stigmatizing language, followed by items focused on drug use history, OUD treatment, HIV prevention, and motivational interviewing. The least highly weighted items focused on past medical history, social history, and family history.

## Results

Of the first cohort of students solicited for reflections on the patient panel experience, 100% (*n* = 40) provided a response. The qualitative responses to the patient panel revealed overall student satisfaction with the patient panel and exposure to patients living with OUD. Three themes (Table) emerged from the reflections: the humanity of people who inject drugs (PWID), different paths to recovery, and the impact of stigma on PWID. Every student reflected positively on the experience, with most mentioning the theme of the humanity of the patients and their comprehension of addiction as a disease. Students were also impressed and surprised by the multiple roads to recovery, some including medication and others preferring Narcotics Anonymous. Students were thankful for the exposure to the patients and highlighted the importance of using nonstigmatizing language when talking to and about patients.

**Table.**
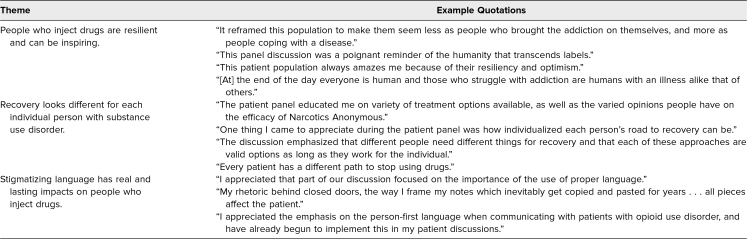
Themes and Quotations From Students’ Patient Panel Reflections

Of the second cohort of students who completed the clinical documentation editing exercise, 100% (*n* = 39) completed both the initial and the edited documentation. For the quantitative test, students’ mean clinical documentation scores before and after the small-group activity and patient panel increased from 10.1 out of 13.5 to 11.3 out of 13.5. There was a significant difference in mean pretest scores (*M* = 10.1, *SE* = 0.28) and mean posttest scores (*M* = 11.3, *SE* = 0.21), *t*(76) = 3.44, *p* < .001.

## Discussion

Given the recent surge in preventable overdose deaths in the US and the syndemic nature of COVID-19 and OUD, our goal of preparing our students to deliver culturally appropriate care to people living with OUD could not be more emergent.^24^ Our curriculum, delivered in the medicine clerkship, resulted in a significant improvement in clinical documentation after completion of the small-group activity and exposure to an interactive patient panel of patients living with OUD. Qualitative feedback from students, provided anonymously, was resoundingly favorable, with most reflection centering on the humanity of the patients in the panel discussion.

Medical education research on OUD and other SUDs has identified deficiencies in student knowledge and preparedness for practice. Among Massachusetts medical students, only 14% felt adequately trained to care for patients with SUD.^25^ A similar number felt comfortable screening for OUD and counseling on MOUD. A variety of approaches for training medical students on OUD have shown success in short-term survey outcomes, including online training modules, student-tailored DATA-2000 waiver training, small-group discussions, and video-based tutorials.^26–28^ In contrast to studies showing positive survey outcomes of educational interventions, we were able to demonstrate effective application of knowledge gained based on improved clinical documentation.

Previous research on medical student experiences with people with SUDs has shown these experiences to be predominantly negative, including seeing the trauma and stigma suffered by PWID.^29^ Our qualitative work uniquely fostered positive experiences with PWID in the patient panel. Students described PWID as being amazing, resilient, and optimistic rather than helpless and victimized. While understanding the negative health care experiences of PWID is important for trainees, we believe learning a message of hope and strength to be equally important.

This exercise is part of our NextGenMD curriculum at the Miller School and is one component of the longitudinal OUD curriculum implemented to equip our graduates with the following core competencies: (1) screen, intervene, and refer individuals with OUD; (2) induce and maintain patients on MOUD; and (3) treat patients suffering with OUD with compassion, dignity, and respect. Initially, we determined that our undergraduate medical curriculum provided variable exposure to screening, diagnosis, treatment, and referral for patients with OUD. The strength of our educational opportunities for students lies in the diverse patient population we serve in Miami-Dade County. The Miller School is home to Florida's first legal SSP, and our partnership with the safety-net mental health hospital in Miami provides robust educational opportunities for our students. We implemented this curriculum to best prepare our students to respond to the modern overdose crisis, which has been worsened acutely by the pandemic.

There are limitations to our study. Implementation could be hindered by the requirement of an existing SP program to execute the OSCE. The Miller School, as home to an SSP, has unique exposure to patients living with OUD eager to participate in medical education. Other institutions wishing to implement this curriculum may not have an embedded harm reduction organization from which to draw patients. Interested institutions could search for local partner SSPs using the North American Syringe Exchange Network.^30^ Additionally, we did not evaluate the entire third-year class (160 students), and the qualitative reflection and clinical documentation evaluations were completed with two different cohorts of students. Whereas the panel opened with standard questions, impact was variable based on student participation. For the qualitative evaluation, we provided representative reflections from students but did not formally code the qualitative analysis. Finally, we did not evaluate the case-based, small-group discussion in our analyses, but we believe that the positive results of the reflection and clinical documentation exercises were affected by completion of the case.

The medicine clerkship provided an acceptable and feasible opportunity to implement a multifaceted, dynamic educational experience for our students with significant immediate impact on their evaluation of patients with OUD. While much of undergraduate medical education is focused on medical knowledge, our educational exercise also provided students with a lesson in humanity that we believe will enhance their skills in responding to the nationwide overdose crisis.

## Appendices


SP Learner Handout.docxSP Case.docxSP Feedback Script.docxCase - Student.docxCase - Facilitator.docxOSCE Rubric for H and P.xlsx
*All appendices are peer reviewed as integral parts of the Original Publication.*
