# Supplementary material for: Opioid Use Disorder Curriculum: Medicine Clerkship Standardized Patient Case, Small-Group Activity, and Patient Panel
Source: MedEdPORTAL. 2022 May 24;18:11248. doi: 10.15766/mep_2374-8265.11248 (PMC9127032; doi:10.15766/mep_2374-8265.11248)
Supplement: Supplementary file 1 — SP Learner Handout.docxSP Case.docxSP Feedback Script.docxCase - Student.docxCase - Facilitator.docxOSCE Rubric for H and P.xlsx [file mep_2374-8265.11248-s001.zip › mep_2374-8265.11248-s001/A. SP Learner Handout.docx]

# Appendix A: LEARNER INSTRUCTIONS

## PATIENT NAME: Zach

**AGE: 26 years old**

CHIEF COMPLAINT: **“**I’m here because my partner wanted me to come in to get subs for heroin.”

**SETTING (PLACE/TIME):** outpatient clinic

**VITAL SIGNS:**

Blood Pressure: 135/78 Temperature: 37.3 C (99.1F) Respiratory Rate: 14

Heart Rate: 76

BMI: 23

# INSTRUCTIONS TO LEARNERS:

1. *Elicit a history from the patient*
2. *Use motivational interviewing skills*
3. *Counsel on treatment options and decide on a plan with the patient*
4. *Patient encounter length is 20 minutes*

# TIP TO LEARNERS:

1. *Avoid stigmatizing and judgmental language*
