# Supplementary material for: Opioid Use Disorder Curriculum: Medicine Clerkship Standardized Patient Case, Small-Group Activity, and Patient Panel
Source: MedEdPORTAL. 2022 May 24;18:11248. doi: 10.15766/mep_2374-8265.11248 (PMC9127032; doi:10.15766/mep_2374-8265.11248)
Supplement: Supplementary file 1 — SP Learner Handout.docxSP Case.docxSP Feedback Script.docxCase - Student.docxCase - Facilitator.docxOSCE Rubric for H and P.xlsx [file mep_2374-8265.11248-s001.zip › mep_2374-8265.11248-s001/B. SP Case.docx]

Appendix B: *MedEdPORTAL* Standardized Patient Case Development Tool

Date: September 17, 2021

Primary Case Author: Jasmine Tomita-Barber

Secondary Case Author: David Serota

Standardized Patient Educator: Sam Sims

Name of Case: Long and Winding Road to Recovery OSCE

Name of educational and or assessment activity: Opioid Use Disorder and Harm Reduction in the Medicine Clerkship: A Standardized Patient Case, Small-Group Activity, and Interactive Patient Panel

Patient Name: Alex

Chief Complaint:  **I’m here because my girlfriend (or boyfriend) wanted me to come in to get subs for** **heroin.”**

Most likely Diagnosis and Differential with rationale from history and/or physical exam:

**Substance use disorder (or opioid use disorder)**

Challenge question: **“What if I relapse again?”**

Domains: Check all that apply

- **X Professionalism**
- **X Communication and Interpersonal skills**
- **X Medical History**
- **X Physical exam**
- **X Shared Decision Making**
- **X Patient Education**
- Clinical Reasoning
- **X Documentation**
- Handoff
- Presentation
- Other:

Type and level of learner: **3rd year medical student**

Case Objectives: please list specific objectives for each of the domains you have checked above:

1) Motivational interview skills

2) Counselling on treatment options for opioid use disorder

3) Empathy and avoidance of stigmatization

| SETTING: outpatient, in patient, ED, home, nursing home, rehab, group etc. | outpatient |
| --- | --- |
| PATIENT PROFILE: Information about the “patient” that helps select an SP and helps the learner get an understanding of them as a person. SP will know more information about the patient than learner will ever ask but allows SP to portray a fully developed patient personality. If none of the items below are particulars for the case please write “all may be used.” | |
| Age range | 20s-30s |
| Religious/spiritual background | N/A |
| Sex (e.g., male, female, intersex, transwoman, transman) | Male/he/him/his or Female/she/her/hers |
| Sexual Orientation (e.g., heterosexual, lesbian, gay, bisexual, pansexual, queer, asexual) | Heterosexual |
| Gender expression (e.g., man, woman, gender queer) | Man or woman [female-adapted SP script is in brackets throughout] |
| Race/ethnicity: | N/A |
| Physical description (e.g., BMI, height range) | N/A |
| Physical limitations | N/A |
| Patient appearance (e.g., disheveled, hospital gown, business casual, casual) | Casual |
| Moulage + location (e.g., none, bruises, scars, body piercing, tattoos) | Antecubital fossa track marks |
| Affect (e.g., pleasant, cooperative) | Anxious, slightly irritable. Yawning a few times throughout the encounter. Make occasional eye contact, looks around the room. You are unsure if the provider will be willing or able to help. Not smiling, but not angry or hostile. |
| Family group (e.g., who is family, who they live with) | lives on friend’s couch or in car |
| Education | Dropped out of community college |
| Level of health literacy | Basic |
| Employment, if any - present and past, noting any current stresses | Unable to hold a job, does handy-person work occasionally |
| Home/homeless - type of dwelling, number of stories, owned or rented | lives on friend’s couch or in car |
| Financial situation- any current stresses | Low income, experiencing homelessness, Live with friends or in car |
| Insurance Status (e.g., un/under/insured, public/private, HMO/PPO) | uninsured |
| Habits (i.e., diet, exercise, caffeine, smoking, alcohol, drugs) | Severe opioid use disorder |
| Activities (i.e., hobbies, sports, clubs, friends) | Watching sports, used to play basketball |
| Typical day - what is the usual daily routine | Wakes up in friend’s house, injects heroin, attends handy work job is he [she] has one |

|  | | |
| --- | --- | --- |
| CASE INFORMATION | |  |
| Chief Concern: What the patient will say when greeted by the student. The patient’s primary reason for seeking medical care often stated in his/own words. | “I’m here because my girlfriend [boyfriend] wanted me to come in to get subs for heroin.” |  |
| Additional Concerns: Other, if any, concerns the patient has today (i.e., symptoms, requests, expectations, etc.) that will become part of set agenda. | If asked “why did your girlfriend [boyfriend] want, you to come in” or “tell me more about what brought you in today”  - “My girlfriend [boyfriend] and I have both been using for years, she’s been on all the subs before. She [he] wants to get clean and she wants me to try with her too”  If asked “Tell me what you think about that idea”  - “I’m not convinced, I’ve tried getting clean before and it didn’t work out.” |  |
|  | |  |
| THE PATIENT STORY: The SP will be asked to tell their symptom story and the personal and emotion impact for each of their concerns. You will want to write this is the patient voice. The symptom story should be able to answer this question: “Tell me more about [chief concern/additional concern], starting at the beginning and bringing me up to now.”  The personal context should be able to answer questions concerning the broader personal/psychosocial context of symptoms, especially the patient beliefs/attributions.  The emotional context should be able to ask how are you doing with this, how does this make you feel, how has this affected you emotionally? IMPACT: How has this affected your life? How has this been for your family?  SPs should not recite this entire section, but rather may give pieces of information and then allow students to request follow up before continuing. For example, SP can mention taking “some medication for opioid addiction, but it didn’t work out,” then waiting for the student to inquire before elaborating. Another example: the SP could mention being in the hospital recently, but then only give the details if asked something like “Can you tell me what you were in the hospital for?” | I started using in high school after a friend gave me oxycontin. I slowly went from taking pills to injecting. I tried cutting back on my own for years but it hasn’t worked. I even tried Narcotic Anonymous once, but it was too uncomfortable for me and I started shooting up again right after the meeting. My girlfriend [or boyfriend] has used for a while too and tried all the meds, like methadone and suboxone, but relapsed each time. I tried something like that last year and did pretty well for around 8 months. I finally had a steady job. I was still at my buddies’ place sleeping on the couch, and we always had parties where people used heroin, fentanyl, and whatever was around. I was on the suboxone and ended up using at the parties a few times, but less than I normally would have. When I saw my doctor for a follow up appointment my urine tested positive for buprenorphine, amphetamines, cannabis, and heroin. He said I “wasn’t serious enough about getting clean” and told me to come back when I was going to put in “real” effort. I stopped going to him and started using again. I lost the job I had and everything I worked to build up. My girlfriend [boyfriend] and I still live at friend’s houses or in our car.  About a month ago I got hospitalized for a heart infection (endocarditis) and a joint infection (septic arthritis). They wouldn’t give me any pain meds, the doctors and nurses acted like I was just a junkie that was trying to get high. I started going through bad withdrawal—throwing up, sweats, diarrhea. I left the hospital early and went to a local clinic for some extra antibiotics. My girlfriend [boyfriend] wants to try get clean again because she [he] had a friend die recently from an OD. She [he] kept begging me to come to this appointment. I told her [him] that I tried subs before, and I couldn’t quit. I tried and I just couldn’t. I want to support her [him], because I know how hard it is to quit when you’re around it, but I’m not convinced I can. I keep needing to use more to get the same high. We both used late last night, and I can already feel some of the withdrawal symptoms after just a few hours of not using (yawning, anxious, stomach cramps). |  |
| HISTORY OF PRESENT ILLNESS: Although some of the HPI will be given in the patient’s symptom story, the learners will expand the story during the direct question section. Below describe the detailed history, usually about the chief concern, which the student must develop in order to make a useful assessment of the problem: | |  |
|  | |  |
| Onset (when; gradual or sudden) | Alex started using in high school around when his [her] parents got divorced. At the time he [she] was diagnosed with ADHD and depression. His [her] father was a heavy drinker and would get into frequent physical altercations with Alex’s mom. His [her] mom kicked Alex out of the house. He [she] has not spoken to his family in over a year. He [she] and his girlfriend [boyfriend] currently sleep on friend’s couches, where other people are also using heroin, or they sleep in their car. Since he [she] has needed more heroin to achieve the same high, he [she] has spent less time working and more time  acquiring heroin. |  |
| Setting (what was going on or where was patient when symptoms first noticed?) | Mild withdrawal symptoms began when woke up this morning |  |
| Duration (how long) | Couple hours |  |
| Time relationships (frequency, constant or intermittent) | constant |  |
| Location |  |  |
| Radiation |  |  |
| Quality |  |  |
| Amount | Intensity of 4 |  |
| Aggravated by what | Abstaining from opioids |  |
| Relieved by what | He [she] injects heroin when he [she] feels significant withdrawal symptoms coming on. |  |
| Associated with what | Unable to hold down a consistent job, experiencing homelessness (living on friend’s couch or in a car), lost contact with family |  |
| Attitude (what does the patient think is the problem, and how does he/she feel about it) | He [she] knows his [her] heroin use has caused him spend his money and miss work, resulting in unsteady employment and housing. He [she] feels as though his [her] childhood and history of depression and ADHD contributed to his [her] drug use. He [she] wants to stop using heroin in order to better support his girlfriend [boyfriend] and her [his] journey to recovery. He [she] wants to make a good life for him [her] and his [her] partner but is afraid to “detox” since it is a terrible process and he [she] could “relapse” again. He [she] worries about the stigma of his [her] heroin use and how it is perceived by medical professionals, given his [her] recent experiences with healthcare providers. He [she] came to this visit to primarily appease his girlfriend [her boyfriend] and expects to listen to a few treatment options. |  |
| Overall course | Withdrawal symptoms are getting worse, the longer he [she] goes without using |  |
| REVIEW OF SYSTEMS: Significant positives and negatives | |  |
| fatigue |  |  |
| Dry mouth when using |  |  |
| Stomach cramps, normally has constipation |  |  |
| Anxious, depressed insomnia |  |  |
|  | |  |
| Past medical history |  |  |
| Medication allergies (Name and reaction) | N/A |  |
| Environmental allergies (Name and reaction) | N/A |  |
| Illnesses | Diagnosed with depression and ADHD.  Mood is depressed, energy is low, concentration poor, insomnia, anxiety |  |
| Vaccinations | COVID-19 |  |
| Surgeries | N/A |  |
| Accidents/ injuries/ trauma | N/A |  |
| Hospitalization | Hospitalization for heart infection (endocarditis) and joint infection (septic arthritis) a month ago. |  |
|  | |  |
| Inclusive sexual and reproductive history | |  |
| Sexual practices  Sexual partners  Protection: Use of safer sex practices  Use of birth control if appropriate  Risk of intimate partner violence | Never been tested for STDs, tests were drawn at the hospital, but he [she] never knew the results  One current female [male] partner  4 lifetime sexual partners  Feels safe at home  Occasional condom use |  |
| Medications | He has been on MOUD in the past |  |
| Tobacco products:   - Cigarettes - Cigar - Pipe - Chew   E-cigarettes | - **X Never** - Past- year started/year quit - Current   - Quantity   # of years |  |
| Alcohol   - **X Beer** - **X Wine** - **X Liquor** - Other | - Never - Past- year started/year quit - Current   - Quantity - **X Many years** |  |
| Drugs   - **X Weed** - **X Cocaine** - **X Heroin** - Meth - Other - **X IV** - Inhalants - Other | - Never - Past- year started/year quit - **X Current**   - Quantity   - Many years |  |
| Diet (describe) | Does not each much, especially when using heroin |  |
| Exercise (describe) | Sometimes plays basketball with friends |  |
| List any other important social history or information important to this case | Insomnia ~ 5 hr a night  Housing instability, unemployment  Never smoker  Socially, drinks ~7 drinks when partying with  friends  Heroin, fentanyl  Marijuana occasionally  History of crushing and snorting ADHD medication in high school, uses it occasionally when available |  |
| Family history | Father- Alcohol use disorder, died of liver cirrhosis and physically abused Zach’s mother  Mother- Unsure, has not spoken to her in over a year |  |
| Mother, Father, Siblings, Grandparents, and other significant findings. | Has a brother and sister, he has been too embarrassed to reach out to them |  |
|  |  |  |
| Physical Exam- List exam maneuvers expected for this case and any abnormal findings that SP will simulate. (tenderness, hyper-hypo reflex, rebound, weakness etc.) | Dermatologic examination—track marks added with makeup |  |
| PHYSICAL EXAM FINDINGS |  |  |
| Written in layman’s terms: Track marks on forearms | |  |
| General appearance- affect, appearance, position of patient at opening (i.e. sitting, laying down, holding abdomen etc.) | sitting |  |
| 1. Vital signs | T 37.3  RR 14  Heart Rate: 76  BP 135/73  BMI: 23 |  |
| 1. DIAGNOSIS AND DIFFERENTIAL | Severe opioid use disorder, substance use disorder |  |
| 1. Diagnosis with support from positive and negative history and PE findings | Major depressive disorder, cellulitis of antecubital fossa |  |
| 1. Differential with support from positive and negative history and PE findings |  |  |
|  |  |  |
| MANAGEMENT OR DIAGNOSTIC PLAN | Start buprenorphine  Screen for HIV, hepatitis C and STIs |  |
|  | Harm Reduction counseling |  |
|  |  |  |
|  | Safer sex counseling |  |
|  |  |  |
