# Supplementary material for: Opioid Use Disorder Curriculum: Medicine Clerkship Standardized Patient Case, Small-Group Activity, and Patient Panel
Source: MedEdPORTAL. 2022 May 24;18:11248. doi: 10.15766/mep_2374-8265.11248 (PMC9127032; doi:10.15766/mep_2374-8265.11248)
Supplement: Supplementary file 1 — SP Learner Handout.docxSP Case.docxSP Feedback Script.docxCase - Student.docxCase - Facilitator.docxOSCE Rubric for H and P.xlsx [file mep_2374-8265.11248-s001.zip › mep_2374-8265.11248-s001/C. SP Feedback Script.docx]

# Appendix C: CHECKLIST GUIDELINES/Feedback Script

| **GUIDELINES FOR FEEDBACK** |  |
| --- | --- |
| Logistics: | 5 minutes for debrief after SP encounter |
| Technique to be used: | Debriefing with Good Judgment |
| Discussion questions/topics: | How did that go?  What was challenging about approaching the topic of treatment? What would you have done differently? |

Checklist guidelines are a description of the intent of a checklist item. Not all items on a checklist must be included; however, clarification of certain items may be useful for raters/SPs.

This includes specifics of what raters/SPs should be looking for to receive credit for an item. Include examples of questions or approaches a student might take and the appropriate response.

Examples (note these are institution specific, authors do not intend example criteria to serve as recommendations for a specific technique)

## History

- **#1 Learner asks about the patient’s thoughts on their substance use**

Yes/No

Note to scorer: questions about the patient’s perspective, thoughts, or attitude about their own substance use warrants credit. A question only about their significant other’s or family’s perspective of their substance use does not warrant credit.

## #2 Learner asks if the patient ever thought about stopping substance use.

Yes/No

Note to scorer: telling the patient that they “should” stop using or asking why they “haven’t” stopped using yet, does not warrant credit.

## #3 Learner asks if the patient ever experienced withdrawal symptoms.

Yes/No

## #4 The learner asks for permission to discuss treatment options with the patient

Yes/No

Note to scorer**:** questions such as “would it be okay if we talked about possible treatment options” “would you like to discuss treatment options” “may I ask you about your thoughts on different treatment options” “ would you like me to share some ideas about treatment options” warrants credit. Discussing treatment options, without asking permission first, does not warrant credit.

## #5 Learner asks if the patient had ever tried treatment for substance use in the past

Yes/No

## #6 Learner asks if the patient ever considered that an alternative treatment would be effective

Yes/No

## #7 Learner asks how confident the patient feels about carrying out the treatment plan

Yes/No

Note to scorer**:** any questions asking how the patient feels about the treatment plan (comfort level, confidence, etc.) warrants credit.

## #8 Learner What would help me achieve my treatment goals

Yes/No

## #9 Learner discussed my treatment goals

Yes/no

Note to scorer: Discussing medication treatment options such as methadone, buprenorphine-naloxone, naltrexone warrants credit. Also mutual support groups, individual psychotherapy, and group psychotherapy warrants credit. Defining treatment goals for the patient without first asking what their goals are does not warrant credit.

## #10 Learner discussed my treatment options

Yes/no

Note to scorer: Defining treatment goals for the patient without first asking what their goals are does not warrant credit.

## Communication

- **#11 Learner avoided stigmatizing language such as “addict”, “injection drug user”, “substance abuse”, “clean and sober”, “relapse”, “detox”**

Yes/no

Note to scorer: words such as “person with substance use disorder” instead of “addict” or “injection drug user”, and “substance use disorder” instead of “substance abuse”, and “drug free/ free from illicit or nonprescribed medications” instead of “clean/sober”, and “recurrence of substance use” instead of “relapse”, and “medically supervised withdrawal” instead of “detox”.

## #12 Learner asked open ended questions

Yes/No

## #13 Learner spoke to the patient as an equal and partner in their care. Never “talked down” or made the patient feel bad about their decisions

Yes/No

Note to scorer: outlining the treatment plan without patient input does not warrant credit.

## #14 Learner demonstrated empathy

Yes/No

## #15 Learner asked questions in a nonjudgmental manner

Yes/No
