# Supplementary material for: Opioid Use Disorder Curriculum: Medicine Clerkship Standardized Patient Case, Small-Group Activity, and Patient Panel
Source: MedEdPORTAL. 2022 May 24;18:11248. doi: 10.15766/mep_2374-8265.11248 (PMC9127032; doi:10.15766/mep_2374-8265.11248)
Supplement: Supplementary file 1 — SP Learner Handout.docxSP Case.docxSP Feedback Script.docxCase - Student.docxCase - Facilitator.docxOSCE Rubric for H and P.xlsx [file mep_2374-8265.11248-s001.zip › mep_2374-8265.11248-s001/E. Case - Facilitator.docx]

Getting Back on Track

Despite a Painful Back

Objectives of Small Group Case:

1. Explain how to diagnose and treat an opioid overdose
2. Interpret urine drug screening
3. Describe strategies to treat pain in hospitalized patients with opioid use disorder
4. Discuss the pros and cons on different interventions for injection drug use-associated infections
5. Explain how to balance the risk/benefit of applying gold standard treatments to PWID
6. Describe the principle of “harm reduction” and
7. List harm reduction interventions that can be implemented for hospitalized people who inject drugs.

Pre-Listening and reading:

- Weimer MB, Chan CA, Taranto NT, Gorth DJ, Williams PN, Brigham SK, Okamoto E, Watto MF. “#224 Hospital Addiction Medicine”. *The Curbsiders Internal Medicine Podcast.* [https://thecurbsiders.com/episode-list](https://nam10.safelinks.protection.outlook.com/?url=https%3A%2F%2Fthecurbsiders.com%2Fepisode-list&data=04%7C01%7Chetookes%40med.miami.edu%7C0009ddb9e98542bb65d008d979c0c747%7C2a144b72f23942d48c0e6f0f17c48e33%7C0%7C0%7C637674691160779864%7CUnknown%7CTWFpbGZsb3d8eyJWIjoiMC4wLjAwMDAiLCJQIjoiV2luMzIiLCJBTiI6Ik1haWwiLCJXVCI6Mn0%3D%7C1000&sdata=O1nqdqOEstztdDdh%2FGglN55FyQH%2FKWT7%2BOMdFnXNm3Y%3D&reserved=0) Original Air Date July 13, 2020.
- Visconti AJ, Sell J, Greenblatt AD. Primary Care for Persons Who Inject Drugs. *Am Fam Physician*. 2019;99(2):109-116.

Part A

Ms. R is a 36-year-old woman who presents with 10 days of fever and low back pain. For the last 3 days she had to call out from work and has had progressive difficulty getting out of bed to do much of anything on account of severe back pain, fatigue, and fever.

She has a past medical history of anxiety, PTSD related to sexual abuse, and fibromyalgia. She grew up in Miami, works at the local grocery store, and lives with her girlfriend in an apartment. She smokes 3 cigarettes per day, has 2 glasses of wine on the weekend, and does not use illicit drugs. Her home medications include sertraline, pregabalin, clonazepam, and she has been taking ibuprofen for the last few days.

The patient’s vital signs and labs indicate sepsis and her blood cultures drawn last night show Methicillin-Resistant *Staphylococcus aureus* (MRSA). She has received a dose of vancomycin and cefepime and is getting a second liter of lactated ringers bolused intravenously. She received hydromorphone 1 mg for severe pain. Urine drug screen shows benzodiazepines.

When you open the door to the patient’s room in the pre-admission area of the ER, she is disconnected from the monitor, breathing at < 10 breaths per minute, not responsive to verbal stimuli. Pupils are pinpoint and her pulse oximeter is not picking up a good waveform but intermittently show 85%. A small plastic bag of white powder and syringe are seen in the patient’s sheets.

1) Describe signs and symptoms of an opioid overdose

- Hypoventilation – hallmark
- Shallow breathing
- Altered mental status, lethargy
- Miotic pupils (constricted)
- Low heart rate and blood pressure (both can also be normal)
- Decreased bowel sounds

2) What are the next steps you should take in treating opioid overdose? Hint: think Basic Life Support.

- ABCs: airway, breathing, circulation
- If RR ≥12 and O2 sat ≥90% on room air, observe (capnography useful here)
- Provide supplementary oxygen, bag-mask ventilation, or endotracheal intubation depending on response to naloxone
- Give initial IV, IM, or nasal naloxone. Repeat frequently, titrating to respiration rather than mental status.
- If hypoventilation returns, consider naloxone infusion (“drip”)
- How would you assess response to treatment

3) Which opioids are long-acting? How do long-acting opioids relate to risk for opioid overdose? What precautions should be taken when treating opioid overdose that is caused by long-acting opioids?

- Naloxone is short acting, so long-acting opioids will outlast naloxone
- Methadone, long-acting formulations of morphine, oxycodone, or hydrocodone are longer acting. Immediate release morphine, oxycodone, and hydrocodone are shorter acting. Heroin and fentanyl are shorter acting.
- Recurrent opioid overdose signs/symptoms can occur when the naloxone wears off.
- Patients should be monitored at least 1 hour after their last dose of naloxone

4) Explain how to interpret urine drug testing for opioids and what might explain this patient’s urine drug screen results?

- Urine drug screen (UDS) picks up natural opiates, you need additional testing to get synthetic opioids, give example: synthetic is fentanyl
- Steps to consider
  - Does the UDS detect the substance (or its metabolites) that I’m worried about
  - What is the timing of use to expect a positive test
- See reference: <https://www.mayoclinicproceedings.org/article/S0025-6196(16)30825-4/pdf>
  - Salient points from: Most substances are out of **urine within 3 days**. For some drugs, regularity of use impacts length of time test is positive (see single marijuana use vs daily marijuana use). Fentanyl is lipophilic, so it stays in system longer.
- Salient points from “Metabolic Pathways” (2):
  - Tests for “opiate” is usually a test for morphine: use of codeine, morphine, or heroin will turn it positive
  - Hydrocodone, oxycodone, **fentanyl**, hydromorphone must be tested separately
  - This is most relevant for fentanyl, which requires a separate test

Part B

A rapid response is called, and the patient receives 0.5 mg of IV naloxone with good response.

She starts breathing at >12 breaths per minute and begins to mumble and move her extremities. She is monitored in the intermediate care unit overnight and requires no further naloxone. Upon further discussion with the patient, she admits to daily fentanyl injection for the last 2 years. Previously, she was using the local syringe services program (SSP) for clean syringes, but since COVID-19 she has avoided going out at all because her wife has HIV and has a weakened immune system. She has been reusing her syringes but not sharing with anyone. Over 48 hours the patient is diagnosed with lumbar vertebral osteomyelitis, epidural abscess, and aortic valve endocarditis.

She continues to experience severe low back pain which has been exacerbated by all the doctors and students coming in each morning to examine her and the uncomfortable hospital bed. She is also intermittently experiencing symptoms of opioid withdrawal when her hydromorphone starts to wear off.

1. Describe strategies to treat pain in hospitalized patients with opioid use disorder

- Important to treat both opioid withdrawal and acute pain and recognize them as two separate processes (3, 4). Can consult addiction specialist and pain medicine. Advocate for their patients to receive adequate pain control.
- Treatments for opioid withdrawal:
  - Treating withdrawal at the opioid receptor
    - Methadone
    - Buprenorphine
- Treating other systemic effects of opioid withdrawal

| Muscle aches | NSAIDS |
| --- | --- |
| Nausea/vomiting | Anti-emetics |
| Diarrhea | Loperamide |
| Abdominal cramps | Dicyclomine |
| General adrenergic surge | Clonidine |
| Anxiety | Benzodiazepines |

• Non-opioid pain management options:

- - Scheduled acetaminophen
  - Scheduled NSAIDS
  - Muscle relaxants, anxiolytics to help with fear and anxiety of pain and opioid hyperalgesia
  - Topicals: lidocaine, diclofenac gel
  - Interventions: nerve blocks
  - Early mobility, physical therapy, avoid loss of function

Part C

Ms. R is noted to have moderate aortic insufficiency on echocardiography due to her endocarditis and also has a 1 cm vegetation on one of the valve leaflets. She has evidence elevated jugular venous distension, 2 pillow orthopnea, and mild lower extremity edema. MRI of the lumbar spine shows a 2 cm anterior-posterior epidural abscess tracking 5 cm cranial-caudal.

1. Discuss pros and cons of the following interventions and treatment approaches for Ms. R’s infections

| Intervention | Pros | Cons |
| --- | --- | --- |
| Aortic valve replacement surgery | - Reference for this question (5, 6) - Reduction in heart failure - Reduction in systemic embolization - Increased chance of endocarditis cure | - Patient now with new high-risk nidus of infection - Large operation with severe pain - Prosthetic valves have finite lifespan, mechanical valves require anticoagulation |
| Lumbar spine surgery | - Higher chance of cure - Increased speed of recovery and pain improvement - Less chance of chronic low back pain | • Patient now with new high-risk nidus of infection (depends on whether hardware placed) |
| Placement of a peripherally inserted central catheter (PICC) to complete IV antibiotics at home or skilled nursing  facility | - Shorter hospital stay - Complete treatment in more acceptable environment | - Risk of injection with PICC – discuss when this is a real concern - Potential for less adherence |
| Use of oral antibiotics to  treat her infections | - Shorter hospital stay - Most diverse discharge options - No need for PICC | - Potential for less adherence - Lack of close monitoring for toxicity and response to treatment - More drug-drug interactions |

(FYI…for facilitators: Table is good transition to patient panel. Bring the table above into the questions with the patient panel. Take home message: Tailoring treatment to the individual

patient. )

Part D – *“This was their preparation work for today. Students should have filled in the table on their own before class. No need to go over the table if you’re running short on time.”*

1. What are some harm reduction interventions that can be instituted in the inpatient setting for patients hospitalized with complications of injection drug use?

Use “Primary Care for People who Inject Drugs” as a guide:

Visconti AJ, Sell J, Greenblatt AD. Primary Care for Persons Who Inject Drugs. *Am Fam Physician*. 2019;99(2):109-116.

| Evaluate for complications of injection drug use | - References (6, 8) - STI screening - Hepatitis B and C screening - HIV screening |
| --- | --- |
| Prevent infectious diseases | - Hepatitis A and B vaccination - Pneumococcal and tetanus vaccination - Linkage to HIV pre-exposure prophylaxis - Linkage to SSP |
| Prevent overdose | - Provide naloxone, during hospitalization ideally - Initiate medications for opioid use disorder and link to treatment |
| Address comorbidities and other social determinants of health | - Diagnose and treat other psychiatric conditions - Evaluate and address homelessness - Provide “trauma-informed care” |

**References**

- 1. Weimer MB, Chan CA, Taranto NT, Gorth DJ, Williams PN, Brigham SK, Okamoto E, Watto MF. “#224 Hospital Addiction Medicine”. *The Curbsiders Internal Medicine Podcast.* [https://thecurbsiders.com/episode-list](https://nam10.safelinks.protection.outlook.com/?url=https%3A%2F%2Fthecurbsiders.com%2Fepisode-list&data=04%7C01%7Chetookes%40med.miami.edu%7C0009ddb9e98542bb65d008d979c0c747%7C2a144b72f23942d48c0e6f0f17c48e33%7C0%7C0%7C637674691160779864%7CUnknown%7CTWFpbGZsb3d8eyJWIjoiMC4wLjAwMDAiLCJQIjoiV2luMzIiLCJBTiI6Ik1haWwiLCJXVCI6Mn0%3D%7C1000&sdata=O1nqdqOEstztdDdh%2FGglN55FyQH%2FKWT7%2BOMdFnXNm3Y%3D&reserved=0) Original Air Date July 13, 2020.
- 2. Moeller KE, Kissack JC, Atayee RS, Lee KC. Clinical Interpretation of Urine Drug Tests: What Clinicians Need to Know About Urine Drug Screens. Mayo Clin Proc.

2017;92(5):774-96.

- 3. Kale N. Urine Drug Tests: Ordering and Interpreting Results. Am Fam Physician. 2019;99(1):33-9.
- 4. Raub JN, Vettese TE. Acute Pain Management in Hospitalized Adult Patients with Opioid Dependence: A Narrative Review and Guide for Clinicians. J Hosp Med. 2017;12(5):375-9.
- 5. Alford DP, Compton P, Samet JH. Acute pain management for patients receiving maintenance methadone or buprenorphine therapy. Ann Intern Med. 2006;144(2):127-34.
- 6. Serota DP, Vettese T. New Answers for Old Questions in the Treatment of Severe Infections from Injection Drug Use. J Hosp Med. 2019;14:E1-E7.
- 7. Serota DP, Chueng TA, Schechter MC. Applying the Infectious Diseases Literature to People who Inject Drugs. Infect Dis Clin North Am. 2020;34(3):539-58.
- 8. Theisen-Toupal J, Ronan MV, Moore A, Rosenthal ES. Inpatient Management of Opioid Use Disorder: A Review for Hospitalists. J Hosp Med. 2017;12(5):369-74.
- 9. Visconti AJ, Sell J, Greenblatt AD. Primary Care for Persons Who Inject Drugs. *Am Fam Physician*. 2019;99(2):109-116.
